# The need to redefine diabetic cardiomyopathy as a unique clinical entity that requires pharmacotherapy

**DOI:** 10.3389/jpps.2025.15503

**Published:** 2025-09-23

**Authors:** Alexa N. King, John R. Ussher

**Affiliations:** ^1^ Faculty of Pharmacy and Pharmaceutical Sciences, University of Alberta, Edmonton, AB, Canada; ^2^ Alberta Diabetes Institute, University of Alberta, Edmonton, AB, Canada; ^3^ Cardiovascular Research Institute, University of Alberta, Edmonton, AB, Canada

**Keywords:** heart failure, diabetic cardiomyopathy, type 2 diabetes, energy metabolism, diastolic dysfunction

The origins of the pathology referred to as diabetic cardiomyopathy (DbCM) can have its roots traced to postmortem findings from Rubler and colleagues in 1972, where they reported left ventricular hypertrophy and congestive heart failure of no known cause in 4 individuals [1]. Furthermore, these individuals demonstrated no overt signs of coronary artery disease, hypertension, or valvular disease. Based on these findings, DbCM is often defined as ventricular dysfunction in the absence of coronary artery disease and/or hypertension [2, 3]. However, in the 50-plus years that have followed this study, preclinical and clinical research have continued to advance the field’s understanding of the pathology of DbCM, and it is becoming clear that DbCM is a clinical entity that stands to benefit from being redefined. Our view on this matter stems from the original terminology not being highly representative of the cardiac phenotype present in people living with diabetes, particularly in those with early-stage type 2 diabetes (T2D). Herein we will elaborate on why DbCM should be redefined, in order to pave the way towards developing pharmacotherapies for specifically treating its pathology.

Advancements in our understanding of DbCM have demonstrated that it is frequently characterized by diastolic dysfunction, though this is often undiagnosed in people with T2D, particularly in the early stages of the disease when routine cardiac screening is not the focus of clinical management [2, 3]. In support of this, recent studies in 18 individuals living with early-onset T2D (mean age 34.9 years and mean diabetes duration 3.1 years) exhibited signs of diastolic dysfunction as assessed using pulse wave and tissue Doppler ultrasound echocardiography (decreased mitral E/A and e′/a′ ratios, increased E/e′ ratio) [4]. Likewise, in a cross-sectional study involving 855 individuals with T2D (mean age 53.4 years and mean diabetes duration 6.0 years), ∼48% demonstrated signs of diastolic dysfunction as determined by a decline in the E/A ratio in the mitral and septal basal regions [5].

It has also become clear through both preclinical and clinical studies that the heart in DbCM is characterized by a distinct metabolic profile. In particular, myocardial fatty acid oxidation rates are increased, whereas myocardial glucose oxidation rates are robustly impaired in DbCM, which has been observed in isolated working heart perfusions from genetic mouse models of T2D (e.g., *ob*/*ob* and *db*/*db* mice) or dietary-induced models of T2D (e.g., high-fat diet feeding plus low-dose streptozotocin administration) [6–8]. While it can be argued that a limitation of these studies are that the heart is perfused *ex vivo* and removed from the true metabolic environment of T2D, *in vivo* studies in humans with T2D using either positron emission tomography imaging or hyperpolarized carbon-13 magnetic resonance spectroscopy have recapitulated these observations [9, 10]. Of translational relevance, several preclinical studies have demonstrated that pharmacologic approaches to overcome impaired myocardial glucose oxidation can alleviate diastolic dysfunction in experimental models of DbCM [7, 8, 11].

Based on the conclusions of these studies, we previously proposed that DbCM be redefined as “diastolic dysfunction in the presence of altered myocardial metabolism in a person with diabetes, but absence of other known causes of cardiomyopathy and/or hypertension” [2]. If therapies are to be developed for the specific treatment of DbCM, a clear definition that can be applied to clinical trial design is necessary in order for applying inclusion/exclusion criteria relating to patient recruitment. Nonetheless, there are limitations with the new definition that we have proposed for DbCM. In particular, this new definition cannot be universally applied to a DbCM phenotype that is truly representative of the diabetic population. While many individuals living with T2D will have a cardiac phenotype aligning with this definition, there will still be individuals with diabetes associated with systolic dysfunction that more strongly resemble a heart failure with reduced ejection fraction phenotype. Similar to what happened with the heart failure classification system, a singular definition of DbCM may prove unsuitable for the field and thus multiple classifications will be necessary. Another limitation with our proposed definition is that it involves ruling out other cardiac pathologies, and a definition/classification that dictates the pathology without needing to rule out other possibilities may be more clinically meaningful with regards to carrying out clinical trials aimed at developing pharmacotherapies for DbCM. Last, our proposed definition is most reliant on observations of preclinical and clinical studies of T2D, but the reality remains that those living with T1D are also at risk of DbCM, and it remains unknown whether this definition would apply to those individuals.

Despite these concerns, an agreed upon revised definition of DbCM that could be more universally applied clinically could have major implications for the management of people living with diabetes. As previously stated, increasing evidence supports that diastolic dysfunction is often present in people living with early-stage T2D [4, 5]. What remains unknown, is what the long-term impact on cardiovascular outcomes are for these individuals if their diastolic dysfunction is clinically managed? Of clinical relevance, people living with T2D are also at increased risk for heart failure with preserved ejection fraction (HFpEF), which is the more prevalent form of heart failure in diabetes and also characterized by diastolic dysfunction. Hence, does successful management of DbCM and its associated diastolic dysfunction decrease future incidence of HFpEF? Without a revised definition of DbCM that can be more easily applied to clinical trial design to facilitate patient recruitment, the required studies needed to answer these questions will prove challenging to pursue and present a roadblock towards developing pharmacotherapies for this disorder.
